# Design and Synthesis of N-Doped Porous Carbons for the Selective Carbon Dioxide Capture under Humid Flue Gas Conditions

**DOI:** 10.3390/polym15112475

**Published:** 2023-05-27

**Authors:** Mahmoud M. Abdelnaby, Mansur Aliyu, Medhat A. Nemitallah, Ahmed M. Alloush, El-Hassan M. Mahmoud, Khaled M. Ossoss, Mostafa Zeama, Moataz Dowaidar

**Affiliations:** 1 Interdisciplinary Research Center for Hydrogen and Energy Storage (IRC-HES), King Fahd University of Petroleum and Minerals (KFUPM), Dhahran 31261, Saudi Arabia; mansur.aliyu@kfupm.edu.sa (M.A.); medhatahmed@kfupm.edu.sa (M.A.N.); ahmed.mohamed3580@alum.kfupm.edu.sa (A.M.A.); g202113350@kfupm.edu.sa (E.-H.M.M.); g202113390@kfupm.edu.sa (K.M.O.); moataz.dowaidar@kfupm.edu.sa (M.D.); 2 Aerospace Engineering Department, King Fahd University of Petroleum and Minerals (KFUPM), Dhahran 31261, Saudi Arabia; 3 SDAIA-KFUPM Joint Research Center for Artificial Intelligence (JRC-AI), King Fahd University of Petroleum and Minerals (KFUPM), Dhahran 31261, Saudi Arabia; 4 Bioengineering Department, King Fahd University of Petroleum and Minerals (KFUPM), Dhahran 31261, Saudi Arabia

**Keywords:** porous polymer, nitrogen-doped carbons, porous carbons, carbon dioxide capture, humid flue gas

## Abstract

The design of novel porous solid sorbents for carbon dioxide capture is critical in developing carbon capture and storage technology (CCS). We have synthesized a series of nitrogen-rich porous organic polymers (POPs) from crosslinking melamine and pyrrole monomers. The final polymer’s nitrogen content was tuned by varying the melamine ratio compared to pyrrole. The resulting polymers were then pyrolyzed at 700 °C and 900 °C to produce high surface area nitrogen-doped porous carbons (NPCs) with different N/C ratios. The resulting NPCs showed good BET surface areas reaching 900 m^2^ g^−1^. Owing to the nitrogen-enriched skeleton and the micropore nature of the prepared NPCs, they exhibited CO_2_ uptake capacities as high as 60 cm^3^ g^−1^ at 273 K and 1 bar with significant CO_2_/N_2_ selectivity. The materials showed excellent and stable performance over five adsorption/desorption cycles in the dynamic separation of the ternary mixture of N_2_/CO_2_/H_2_O. The method developed in this work and the synthesized NPCs’ performance towards CO_2_ capture highlight the unique properties of POPs as precursors for synthesizing nitrogen-doped porous carbons with a high nitrogen content and high yield.

## 1. Introduction

Global warming has become one of the main threats to human life because of the massive amounts of CO_2_ being released into the atmosphere. The emitted CO_2_ is mainly a result of human activity and burning fossil fuels to produce the energy necessary for many industries that improve our life welfare. Accumulation of CO_2_, a heat-trapping greenhouse gas in the atmosphere, is causing a continuous increase in Earth’s temperature, which is altering the climate and could, if not controlled, lead to the end of life on Earth [1,2,3]. To handle this global catastrophe, many researchers are racing discover new methods and technologies, especially for CO_2_ capture, which may give the world a fighting chance of survival [4]. Currently, amine scrubbing is the most well-developed method available for capturing CO_2_ from fossil fuels [5]. Even though this method is well-understood and widely used in industries, it still has some significant drawbacks, such as its large energy consumption, corrosion problems, and solvent degradation into possibly harmful materials. So, to avoid these issues, an up-and-coming alternative method is the implementation of porous solid sorbents that can adsorb CO_2_ selectively under ambient conditions [6,7]. Many scientific findings have proven that porous materials with flexible arrangements for pressure, temperature, and vacuum swing adsorption are considered the most promising for CO_2_ capture and sequestration (CCS) [8,9]. There are multiple families of materials that can be included under the title of porous solid sorbents; these materials include zeolites, metal–organic Frameworks (MOFs) [10,11,12], porous organic polymers (POPs) [13,14,15], and porous carbons (DPC) [16,17,18]. N-doped porous carbons (NPCs) have garnered much attention in this field of study due to the controllability of their pore architecture, shape or size for any targeted application [19,20], ease of preparation, sizeable variety of raw materials, and wide-ranging applications [21,22]. Although porous carbons have been proven to have high CO_2_ capacities, achieving high CO_2_/N_2_ selectivity is challenging due to the weak surface chemical interaction. Adding CO_2_-philic moieties, such as nitrogen atoms in NPCs, within the carbon framework can improve their adsorption energy, increasing CO_2_ uptake and CO_2_/N_2_ selectivity. This is the primary purpose of nitrogen-doping, which will produce greater affinity towards CO_2_ because of the strong interaction between the basic N-functional sites and the acidic CO_2_ molecules [23,24].

Here, we have developed a series of new NPCs derived from melamine pyrrole-based POPs. The pyrolysis of the melamine pyrrole-based POPs produced a high yield compared to other porous carbon sources. The developed porous carbon enhanced the CO_2_ capture performance compared to the parent POPs.

## 2. Materials and Methods

All chemicals and reagents were used as received without any purification or modification. Melamine (99% purity), p-formaldehyde (99% purity), and pyrrole (99% purity) were obtained from Alfa Aesar, Hyderabad, India. Hydrochloric acid (37 wt.%; HCl) and toluene (99% purity) were brought from Acros Organics, Geel, Belgium. Methanol (99.9% purity; MeOH), *N*,*N*’-dimethylformamide (99% purity; DMF), and palladium on carbon (5% *w*/*w*; Pd/C) were obtained from Sigma Aldrich, Missouri, USA. Ultrapure water was prepared using a milli-Q ultrapure water purification system. For gas sorption analysis, nitrogen gas (99.999% purity), carbon dioxide gas (99.99% purity), helium (99.999% purity), and zero air (99.999% purity) were provided by Air Liquid Co. Dammam, Saudi Arabia.

Fourier transform infrared (FT-IR) spectra were recorded with a PerkinElmer 16 PC spectrometer using potassium bromide (KBr) pellets. The output spectral bands were labeled as s, strong; m, medium; w, weak; and br, broad. The spectra were recorded over 4000–400 cm^−1^ in transmission mode. A solid-state carbon-13 nuclear magnetic resonance (^13^C NMR) analysis was performed on a Bruker 400 MHz instrument, operating at 125.65 MHz at room temperature. Cross-polarization magic angle spinning (CP-MAS) at 14 kHz was used. Powder X-ray diffraction (PXRD) patterns were collected using a Rigaku MiniFlex II X-ray diffractometer (Tokyo, Japan) with a Cu Kα radiation source (λ = 1.54178 Å). On a Quantachrome Quadrasorp instrument, low-pressure nitrogen gas sorption isotherms were measured. The experiments were carried out in a bath of liquid nitrogen at 77 K. Measurements at 273 and 298 K were managed using a water bath connected to an adjustable chiller. Thermogravimetric Analysis (TGA) was measured with a TA Q500 instrument. Measurements were made under airflow (20 mL/min) and a heating rate of 10 °C/min.

### 2.1. Polymers Synthesis

#### 2.1.1. POP101

Synthesis was achieved following a previously reported method [23]. Melamine (1.26 g, t mmol) and p-formaldehyde (1.80 g, 60 mmol) were added to DMF (50 mL) inside a 250 mL round-bottomed flask. The mixture was left at room temperature for 5 min under vigorous stirring (700 rpm). Pyrrole (0.67 g, 10 mmol) was then added, and the mixture was stirred wholeheartedly for an extra 5 min. While stirring, HCl (1.0 mL) was added to the reactants slowly, and then immediately, and the flask was sealed with a rubber septum. The reaction mixture was then transferred to a pre-heated oil bath at 90 °C and stirred at the same rate for 24 h. After 24 h, the reaction mixture was removed and left to cool to room temperature. Afterward, the mixture was poured in MeOH (50 mL), closed tightly, and was sonicated for 30 min. The solid product was then filtered and washed in DMF (50 mL) for 1 h. Finally, the resulting solid was further purified using Soxhlet extraction with methanol overnight. The resulting polymer was dried in a vacuum oven at 80 °C for 12 h, yielding a total of (3.53 g) of dry product.

#### 2.1.2. POP102

POP102 was synthesized following the exact procedure as mentioned above, only varying the (melamine: pyrrole) molar ratio to 1:2. Melamine (1.26 g, 10 mmol) and p-formaldehyde (2.70 g, 90 mmol) were added to DMF (70 mL) inside a 250 mL round-bottomed flask. The mixture was left at room temperature for 5 min, under vigorous stirring (700 rpm). Pyrrole (1.40 g, 20 mmol) was then added, and the mixture was stirred wholeheartedly for 5 min. While stirring, HCl (1.0 mL) was slowly added to the reactants; the flask was then immediately sealed with a rubber septum. The reaction mixture was then transferred to a pre-heated oil bath at 90 °C, and kept stirring at the same rate for 24 h. After 24 h had passed, the reaction mixture was removed and left to cool to room temperature. Afterward, the mixture was poured in MeOH (50 mL), closed tightly, and sonicated for 30 min. The solid product was then filtered and washed in DMF (50 mL) for 1 h. Finally, the resulting solid was further purified using Soxhlet extraction with methanol overnight. The resulting polymer was dried in a vacuum oven at 80 °C for 12 h, yielding a total of (4.94 g) of black solid, dense particles.

#### 2.1.3. POP103

POP103 was synthesized following the exact procedure as mentioned above, only varying the (Melamine: pyrrole) molar ratio to 1:3. Melamine (0.63 g, 5 mmol) and p-formaldehyde (1.80 g, 60 mmol) were added to DMF (50 mL) inside a 250 mL round-bottomed flask. The mixture was left at room temperature for 5 min under vigorous stirring (700 rpm). Pyrrole (1.01 g, 15 mmol) was then added, and the mixture was stirred wholeheartedly for an extra 5 min. While stirring, HCl (1.0 mL) was slowly added to the reactants; the flask was then immediately sealed with a rubber septum. The reaction mixture was then transferred to a pre-heated oil bath at 90 °C and stirred at the same rate for 24 h. After 24 h had passed, the reaction mixture was removed and left to cool to room temperature. Afterward, the mixture was poured into MeOH (50 mL), closed tightly, and sonicated for 30 min. The solid product was then filtered and washed in DMF (50 mL) for 1 h. Finally, the resulting solid was further purified using Soxhlet extraction with methanol overnight. The resulting polymer was dried in a vacuum oven at 80 °C for 12 h, yielding a total of (2.87 g) of dry final product.

#### 2.1.4. POP104

POP104 was synthesized following the exact procedure as mentioned above, only varying the (melamine: pyrrole) molar ratio to 1:4. Melamine (0.63 g, 5 mmol) and p-formaldehyde (2.25 g, 75 mmol) were added to DMF (50 mL) inside a 250 mL round-bottomed flask. The mixture was left at room temperature for 5 min, under vigorous stirring (700 rpm). Pyrrole (1.34 g, 20 mmol) was then added, and the mixture was stirred wholeheartedly for an extra 5 min. While stirring, HCl (1.0 mL) was slowly added to the reactants; the flask was then immediately sealed with a rubber septum. The reaction mixture was then transferred to a pre-heated oil bath at 90 °C, and kept stirring at the same rate for 24 h. After 24 h had passed, the reaction mixture was removed and left to cool to room temperature. Afterward, the mixture was poured into MeOH (50 mL), closed tightly, and sonicated for 30 min. The solid product was filtered and washed in DMF (50 mL) for 1 h. Finally, the resulting solid was further purified using Soxhlet extraction with methanol overnight. The resulting polymer was dried in a vacuum oven at 60 °C for 12 h, yielding a dense black precipitate with a weight of 4.17 g.

### 2.2. N-Doped Carbon Synthesis

Carbonization of the polymers was carried out using a horizontal quartz tubular reactor with a continuous nitrogen flow. The process begins with 1.0 g of polymer being heated gradually, rate = 5.0 °C/min, up to 360 °C. After the temperature ramp, an isotherm is maintained at this temperature for 3 h. Afterward, the temperature is raised to pyrolysis temperature (700 °C or 900 °C) at a rate of 3 °C/min and held for an additional hour. Finally, the material was left to cool down to room temperature.

**Figure 1:**
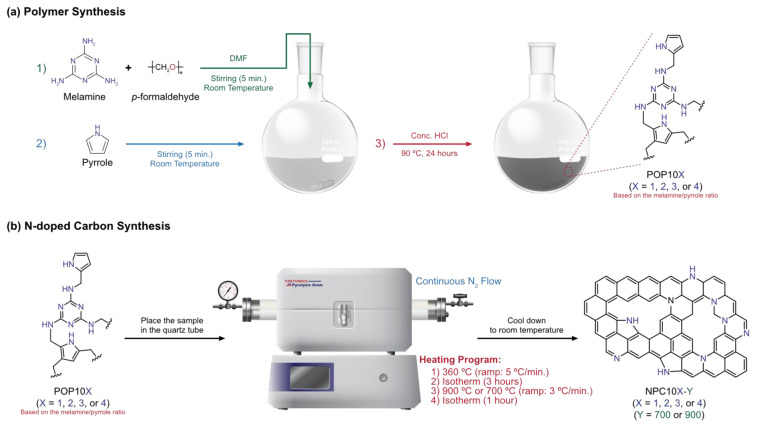
Detailed synthetic scheme of the melamine pyrrole-based POPs (**a**), and their pyrolysis to NPCs (**b**).

In addition to the detailed synthetic description provided for polymers and NPCs, we have summarized both of the synthetic procedure and the synthetic conditions in Figure 1 and Table 1 respectively.

## 3. Results and Discussion

The main goal of this work is to develop a facile and practical method to prepare a unique nitrogen-doped porous carbon material for the selective capture of CO_2_. Our two-step synthetic strategy helped us gain control over the nitrogen content within the final NPCs by optimizing the porous material preceding the pyrolysis, which is the crosslinking of melamine and pyrrole with a methylene linkage. Both melamine and pyrrole are aromatic amine-based, CO_2_-philic monomers. By varying their molar ratios concerning each other, we obtained various versions of the same framework POP, but with varying degrees of nitrogen content within the framework. Furthermore, the monomer ratio optimization enables the tuning of the micro/mesopore nature of the resulting framework. Increasing the ratio of pyrrole, which has four crosslinking points, increases the degree of crosslinking and rigidity. Utilizing the POPs generated from this optimization technique is crucial for guaranteeing the obtainment of the optimum NPC material for the targeted application.

The successful synthesis of the polymers and the NPCs was proven with different characterization techniques. Their physical properties were studied to evaluate how these materials can perform and compete against other materials in CO_2_ capture. Although various NPCs have been synthesized from different POPs and at different temperatures, we selected the best-performing material (NPC10X-900) for the structural characterization, referred to as NPC10X, without reflecting the temperature.

### 3.1. Structural Characterization & Permanent Porosity

**Figure 2:**
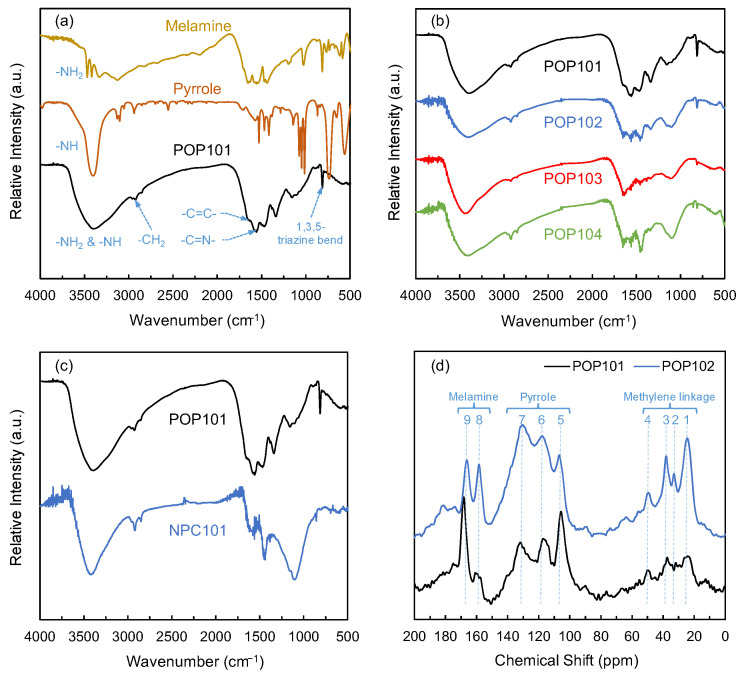
(**a**) FT-IR spectra of the melamine, pyrrole, and POP101; (**b**) FT-IR spectra of POP101, POP102, POP103, and POP104 stacked together for clear comparison; (**c**) FT-IR spectra of POP101 and its corresponding NPC101; (**d**) Solid-state ^13^C NMR for POP101 and POP102 polymers.

FT-IR analysis was used to spot the changes in materials due to any alterations in their functionalities. Both pure monomers’ spectra, pyrrole and melamine, were graphed in comparison with the synthesized polymers to facilitate the comparison of the fingerprints between their FT-IR spectra (Figure 2). Figure 2a shows a clear difference between the monomers and the polymer POP101 at high wavenumber. At ~3500 cm^−1^, we can identify the two characteristic bands of a primary amine in melamine. In pyrrole, only one band appears in approximately the same wavenumber corresponding to the secondary amine stretch. At the same time, in the case of POP101, broadband reflects the presence of both amine types within different environments in the polymer. All three other polymers also exhibited similar behavior with a broad absorption band around 3413 cm^−1^ (Figure 2b). A shoulder to the absorption band at around 3240 cm^−1^ proves that the POPs contained free amine moieties. Additionally, the presence of the aromatic -C=C- vibrational bands of the pyrrole, located at 1515 cm^−1^, in the POP101 spectrum provide strong evidence that this monomer is incorporated in the polymer [23]. Finally, the presence of the melamine monomer was indicated by the presence of the characteristic out-of-the-plane bend of the 1,3,5-triazine ring at 812 cm^−1^ [25]. The ^13^C NMR proves the monomers’ successful incorporation and the aliphatic linkage formation (Figure 2d). The structure elucidation matches the previous study’s matching chemical shifts [23]. The range between 20–50 ppm shows around four main peaks representing the formation of the methylene linkage through different monomer connections. The peaks of pyrrole carbons are shown within a range of 100–140 ppm, while the peaks at 150–170 ppm represent the 1,3,5-triazine carbons.

**Figure 3:**
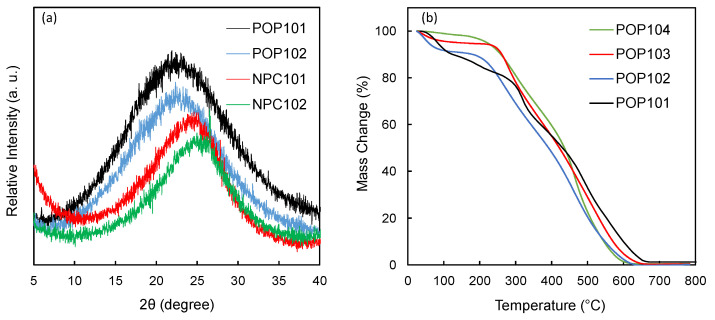
(**a**) Thermogravimetric analysis (TGA) of the POPs under air atmosphere. (**b**) Stacked PXRD patterns of the synthesized polymers POP101, POP102 and their corresponding NPCs, NPC101 and NPC102. Measurements were made at a scan rate of 2 degrees/min.

The crystallinity of the materials was investigated with PXRD, and the produced patterns show almost identical behavior with a broad peak centered around 20–25 degrees, indicating their amorphous nature, with no evidence of any long-range order or repeating atomic structure for both POPs and NPCs (Figure 3a). The thermal stability of the polymers was investigated with TGA, as shown in Figure 3b, and all polymers showed good stability up to ~250 °C.

**Figure 4:**
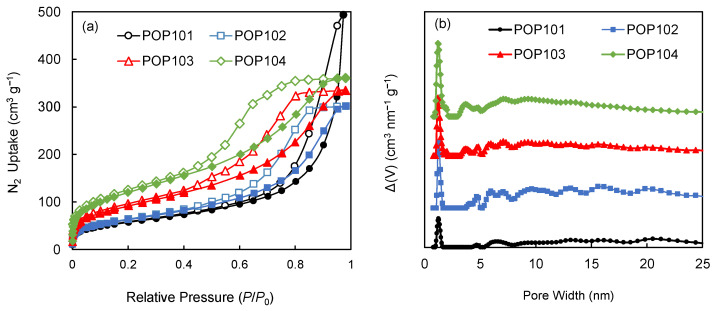
(**a**) Low-pressure N_2_ isotherms measured at 77 K, solid and hollow markers represent the adsorption and desorption branches respectively; (**b**) pore size distribution (PSD) of the parent porous organic polymers (POPs) as calculated using the quenched solid density functional theory (QSDFT) with N_2_ at 77 K on a carbon model (slit/cylindrical/sphere pores, adsorption branch). POP101 (black circles), POP102 (blue squares), POP103 (red triangles), and POP104 (green rhombuses). The markers represent the experimental data points, while the connecting lines just serve to guide the eye.

Measurement of a low-pressure N_2_ isotherm at 77 K was conducted to examine the architectural stability and permanent porosity of the synthesized POPs and NPCs (Figure 4a). The isotherms obtained from POP102, POP103, and POP104 all showed a behavior characteristic of Type-IV isotherms according to the classification system of the International Union of Pure and Applied Chemistry (IUPAC) [26]. An initial uptake at very low *P*/*P*_0_, followed by a slight increase up to ~0.5 *P*/*P*_0_, then a final increase corresponding to the condensation within the mesopores, which ends in either saturation or an inflection point, then forms a hysteresis loop upon desorption. For POP101, the isotherm started exactly like the other polymers, but showed a different pattern at high *P*/*P*_0_, with a very sharp uptake, which makes it a Type-II isotherm. The hysteresis formed upon desorption is potentially caused by the mesopore filling due to capillary condensation/evaporation [27,28].

**Figure 5:**
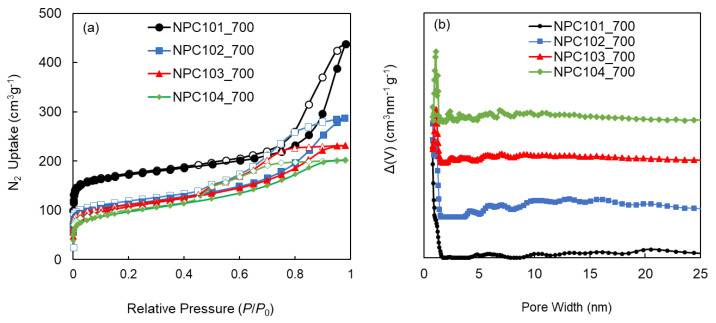
(**a**) Low-pressure N_2_ isotherms measured at 77 K, solid and hollow markers represent the adsorption and desorption branches respectively; (**b**) pore size distribution (PSD) of the N-doped porous carbons (NPCs) as calculated using the quenched solid density functional theory (QSDFT) with N_2_ at 77 K on a carbon model (slit/cylindrical/sphere pores, adsorption branch). NPC101-700 (black circles), NPC102-700 (blue squares), NPC103-700 (red triangles), and NPC104-700 (green rhombuses). The markers represent the experimental data points, while the connecting lines just serve to guide the eye.

Pore size distributions (PSD) were calculated to obtain an idea of the most common pore sizes, their distribution, variation, and how the different pores contribute to the total pore volume of the material. All PSDs are calculated using the quenched solid density functional theory (QSDFT) with N_2_ at 77 K on the carbon model (slit/cylindrical/sphere pores, adsorption branch) [29,30]. In Figure 4b, the PSD of the polymers shows a combination of micro-, meso-, and macro-pores with varying quantities from one polymer to another, which is an expected outcome given that the polymers are amorphous. Upon carbonization, all of the NPCs showed a much higher microporous nature, represented in the higher uptake at low *P*/*P*_0_ (similar to aType-I isotherm) and the higher micropore ratio to other pore sizes in the PSD curves (Figure 5a,b). Surface areas were estimated using the Brunauer–Emmett–Teller (BET) model, with the experimental data chosen in the range of *P*/*P*_0_ = 0.01–0.3. Table 2 summarizes the surface areas and pore characteristics of the four POPs and their corresponding NPCs. The surface areas of the materials greatly enhanced after the carbonization; for instance, the BET surface area of POP101 increased from 101 m^2^ g^−1^ to 700 m^2^ g^−1^ for its corresponding NPC101-900. One plausible explanation is that at these high temperatures, various ring fusions or closures could occur [31], which will result in creating more walls and tightening the pore sizes. 

### 3.2. Thermodynamic Uptake Capacity

**Figure 6:**
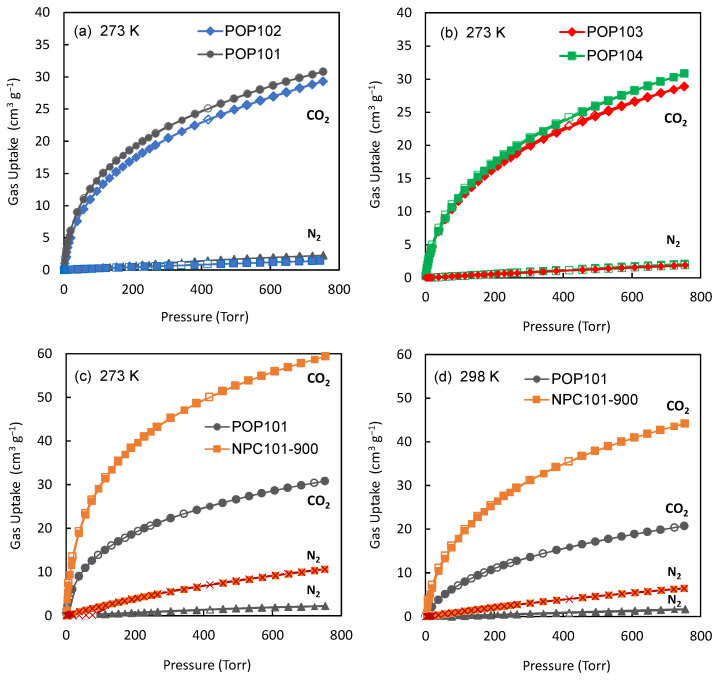
CO_2_ and N_2_ single component gas sorption isotherms of (**a**) POP101 and POP102 at 273 K, (**b**) POP103 and POP104 at 273 K, (**c**) POP101 and NPC101-900 at 273 K, and (**d**) POP101 and NPC101-900 at 298 K. The markers represent the experimental data points, while the connecting lines just serve to guide the eye.

The improved surface area of the carbonized samples (NPCs) encouraged us to investigate them more. So, we evaluated the thermodynamic gas adsorption capabilities for the synthesized POPs and the two best-performing carbonized samples. Low-pressure, single-component gas adsorption isotherms of CO_2_ and N_2_ gases were measured at 273 K and 298 K (Figure 6). At 273 K and 760 Torr, POP101 and POP102 showed a moderate CO_2_ uptake capacity (30.0 and 29.3 cm^3^ g^−1^, respectively), but the N_2_ uptake capacities under identical experimental conditions were very low (1.2 and 2.2 cm^3^ g^−1^, respectively). In contrast to the N_2_ isotherm, the POPs exhibited a substantially steeper CO_2_ uptake in the low-pressure region, which suggests stronger interactions between polymers and CO_2_ molecules. More interestingly, the CO_2_ uptake capacity of NPC101-900 was nearly twice that of the polymer POP101 (Figure 6c,d). A summary of the CO_2_ sorption data for the POPs and NPCs at different temperatures is provided in Table 3.

### 3.3. Thermodynamic Analysis of the Adsorption Process

The change in entropy (ΔS°), the difference in the enthalpy of reaction (ΔH°), and the change in Gibbs free energy (ΔG°) are the thermodynamic properties considered for the adsorption process. These properties were estimated using Van Hoff’s expression, and are presented in Equations (1) and (2) [32,33,34].

(1) $$\ln K_{F}=\frac{{-∆H}^{{}^{\circ}}}{RT}+\frac{{∆S}^{{}^{\circ}}}{R},$$

(2) $${∆G}^{{}^{\circ}}=-RT\ln K_{F},$$

where *K_F_* is the Freundlich constant, *R* is the universal gas constant, and *T* is the temperature of adsorption (Kelvin). The values obtained for the thermodynamic parameters are shown in Table 4. It can be observed from Table that all the thermodynamic properties have a negative sign. The negative sign in the entropy changes values indicates that the adsorbate molecules are highly ordered. This can also reveal how the CO_2_ molecules behave during the process, as the molecules appear to transition from a random state to a more organized one [34,35]. The NPC101 molecules have higher orderliness than the NPC102. The negative sign in the enthalpy change values indicates that the adsorption process is exothermic, and it can also indicate whether the adsorption process is physical (i.e., physisorption) or chemical (i.e., chemisorption) in nature. Values of around −20 kJ/mol suggest that the adsorption process is physisorption, while values ranging from −80 to −200 kJ/mol imply that the adsorption process is chemisorption [33,34,35,36]. Our data show that both NPC101 and NPC102 capture CO_2_ physically (physisorption). The negative values of ΔG° show that the nature of the adsorption process is exothermic and spontaneous [37]. The values of ΔG° at 273 K are higher than those at 298 K for the two materials, which shows that the adsorption process is favorable at a lower temperature [35]. Considering the three thermodynamic properties presented in Table 4, it can be noticed that the adsorption process is more exothermic and spontaneous with the material NPC101, and its molecules display a high degree of orderliness when compared to the material NPC102. Comparing the thermodynamic properties presented here with what is available in the literature for activated carbon [33,37], it can be observed that NPC101 and NPC102 are good candidates, suitable for physical CO_2_ adsorption through fundamental electrostatic, dipole–dipole, and van der Waals interactions. This is based on the adsorption–desorption isotherms that show no hysteresis loops with CO_2_ uptake, and the values of isosteric heat of adsorption.

Another important thermodynamic property that describes the interaction between an adsorbent and an adsorbate is isosteric heat (*E_st_*). The isosteric heat of adsorption can be expressed as the ratio of limitless small change in adsorbate enthalpy to the limitless small change in quantity adsorbed [38]. It illustrates the intensity of interaction between the adsorbate molecules and the adsorbent’s surface. A Clausius–Clapeyron equation is used to estimate adsorption’s isosteric heat expressed in Equation (3) [32,34].

(3) $$E_{st}={\left|RT\frac{\partial ln(P)}{\partial \left(\frac{1}{T}\right)}\right|}_{m_{ads}},$$

The *E_st_* is obtained from the slope of Equation (3) when ln(*P*) is plotted against the inverse of temperature *1/T* at different CO_2_ uptakes, where *P* is the pressure, and *m_ads_* is the CO_2_ uptake or surface coverage. The values of *E_st_* range between 18.52–26.41 kJ/mol for the materials under consideration. This range of values is further confirmed by knowing that the adsorption process is physisorption [39]. The higher value of *E_st_*, which corresponds to the material NPC101, shows interaction and attractiveness between the surface of the material and the molecules of CO_2_, which ended up filling the micropores of the material [34,40,41].

**Figure 7:**
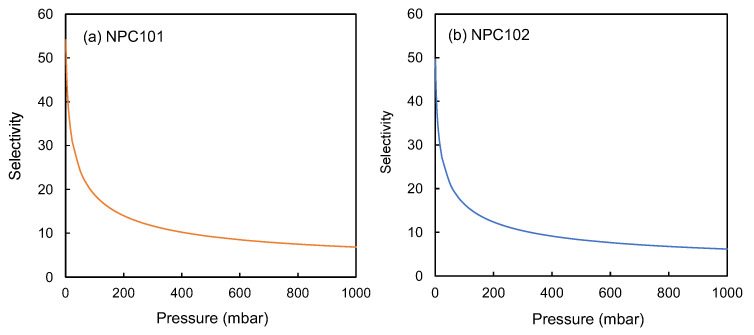
Selectivity plots of CO_2_/N_2_ (20%: 80%) for (**a**) NPC101 and (**b**) NPC102 as calculated by the IAST model at 298 K.

The Selectivity of NPC101 and NPC102 towards the binary mixture of 20% CO_2_ and 80% N_2_ was simulated using the Ideal Adsorption Solution Theory (IAST) model over a varying range of pressures (0–1000 mbar), and the data are presented in Figure 7.

### 3.4. Dynamic CO_2_ Separation

Dynamic breakthrough tests were carried out to assess NPC101-900’s efficacy and selectivity in capturing CO_2_ under real-world flue gas conditions. A typical experiment involves loading an activated sample of NPC101-900 into a bed and exposing it to a gaseous mixture that contains 80% N_2_ and 20% CO_2_. These are volumetric percentages that are meant to mimic the flue gas composition. The full breakthrough capacity of CO_2_ was measured by evaluating the composition ratio of the downstream gas to the feed gas.

(4) $$q_{{co}_{2}}=\frac{1}{m}FCt\left(\frac{298}{273}\right),$$

where $q_{{co}_{2}}$ is the CO_2_ capacity (cm^3^ g^−1^) at 298 K, *m* is the mass of adsorbent (g), *F* is the input flow rate (cm^3^ min^−1^) at STP, *C* is the influent CO_2_ concentration (vol.%), and *t* is the time (min).

**Figure 8:**
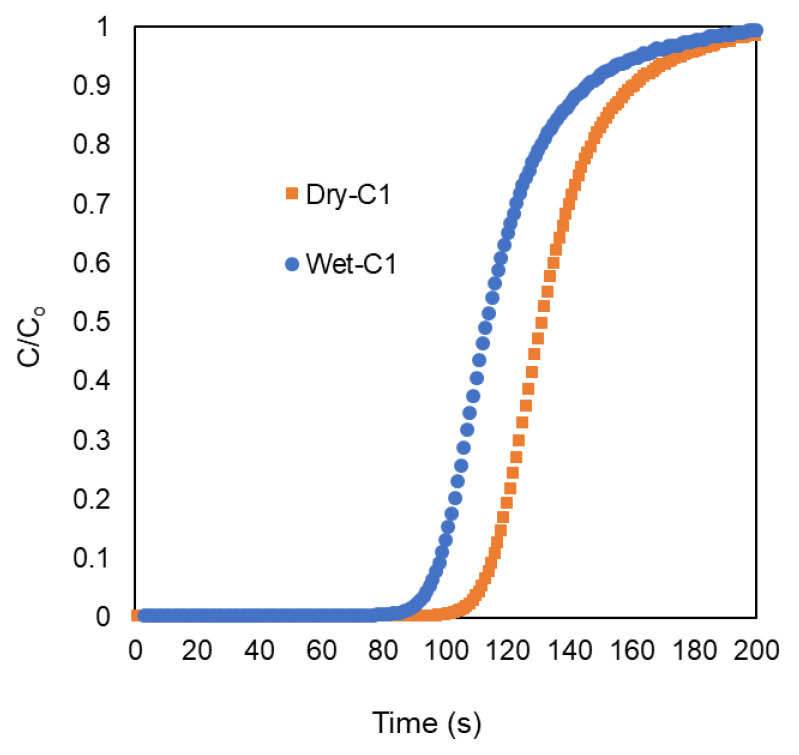
A 20:80 gas mixture containing CO_2_ and N_2_, respectively, under dry (orange squares) or wet (91% RH, blue circles) conditions was flown through a fixed bed of NPC101-900 at 298 K and 1 bar (mass of the sample 0.3 g, and 10 cm^3^ min^−1^ flow rate). Dynamic breakthrough measurements demonstrate the ability of NPC101-900 to separate CO_2_ from N_2_ under wet and dry conditions.

An online mass spectrometer was used to monitor the effluent composition during the experiment to precisely record the breakthrough time (i.e., the moment when a material reaches saturation and extra CO_2_ molecules “break through” the bed). N_2_ gas is the only gas present in the effluent for 1.6 min before CO_2_ reaches its breakthrough point. It is obvious that NCP101-900 selectively retains CO_2_ for a considerable amount of time, even in the wet CO_2_ streams, with a breakthrough time of 1.5 min and a loss of only 10% in the total uptake. These findings show some early promise for NCP101-900, but a crucial factor still needs to be proven. The corresponding dynamic CO_2_ uptake capacity of NCP101-900, computed from the breakthrough time, was 10.6 cm^3^ g^−1^. Since competitive adsorption occurs easily in water, porous materials often struggle to capture CO_2_ selectively. This reduces the material’s ability to absorb CO_2_ and/or makes it less stable and less recyclable over time. We investigated the material’s capability to separate CO_2_ from N_2_ in the presence of water in light of NCP101-900’s original breakthrough results under dry conditions, and investigated its water stability. As a result, NCP101-900 was exposed to a ternary gas mixture that contained CO_2_, N_2_, and H_2_O (20%, 80%, and 91RH, respectively). NCP101-900 was once more capable of selectively retaining CO_2_, as depicted in Figure 8.

**Figure 9:**
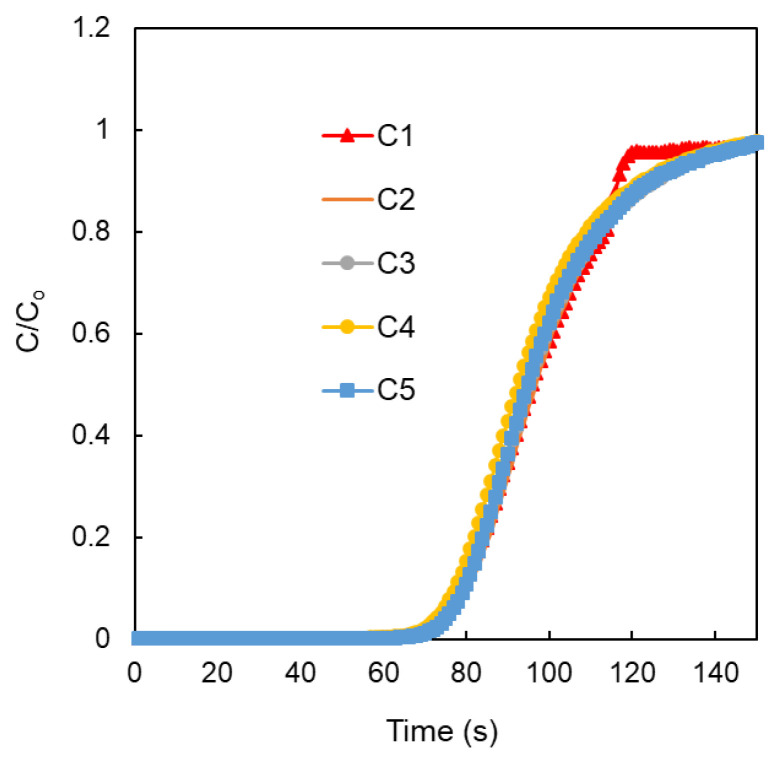
The long-term recyclability performance of NPC101 using wet CO_2_ breakthrough measurements. The desorption/activation of the sample was carried out through N_2_ flow at room temperature. Over complete five consecutive cycles, the dynamic adsorption capacity has not decreased.

The long-term usage and recyclability of an adsorbent material, without performance degradation, is a crucial aspect that must be considered for deployment in an industrial setting. As a result, we performed a continuous multicycle breakthrough measurement (>5 cycles) at 298 K. (Figure 9). NCP101s were first exposed to a wet N_2_ stream (91% RH) for each cycle of this experiment until water saturation was noticed. When the wet N_2_ stream reached saturation, a dry stream of CO_2_ (20% *v*/*v*) was injected, and the effluent was watched for the breakthrough time. Over the course of the multicycle testing, NPC101-900 demonstrated remarkable stability and recyclability. It is crucial to remember that NPC101-900 was regenerated between each cycle by passing a moist N_2_ stream through the material at 298 K. This regeneration process is a notably alluring property for using NPC101-900 as an adsorbent for the selective capture of CO_2_ from actual flue gas mixtures from the perspective of energy costs. Table 5 compares NPCs’ performance with similar related material toward CO_2_ capture.

## 4. Conclusions

We have developed a strategy to investigate and select the best-performing N-doped porous carbon (NPC) prepared from a porous organic polymer (POP). The samples prepared at 900 °C had a higher surface area than those at 700 °C. NPC101-900 has shown a high CO_2_ uptake capacity of 44 cm^2^ g^−1^ at 298 K and 1 bar, and great potential for selectively capturing CO_2_ from binary, CO_2_/N_2_, and ternary CO_2_/N_2_/H_2_O gas mixtures. NPC101-900 showed good dynamic CO_2_ uptake capacities of 10.6 and 10.0 cm^3^ g^−1^ under dry and wet conditions, respectively. Furthermore, withstanding a multicycle continuous breakthrough measurement, in which a subtle regeneration of NPC101-900 was applied at low energy consumption, showed that our material could selectively capture CO_2_ from a wet N_2_ stream over an extended period, without suffering material degradation. The results presented here emphasize a strategy for advancing the synthesis of N-doped porous carbons from porous polymers as prospective and sensible adsorbent materials for industrially relevant and realistic gas separation procedures.

## Figures and Tables

**Table 1:** Summary of the synthetic conditions and optimizations for all POPs and NPCs.

Entry	Material	Precursors	Method	Temperature
1	POP101	Melamine/Pyrrole Molar ratio: 1:1	Solvothermal (DMF)	90 °C
2	POP102	Melamine/Pyrrole Molar ratio: 1:2	Solvothermal (DMF)	90 °C
3	POP103	Melamine/Pyrrole Molar ratio: 1:3	Solvothermal (DMF)	90 °C
4	POP104	Melamine/Pyrrole Molar ratio: 1:4	Solvothermal (DMF)	90 °C
5	NPC101-700	POP101	Pyrolysis	700 °C
6	NPC102-700	POP102	Pyrolysis	700 °C
7	NPC103-700	POP103	Pyrolysis	700 °C
8	NPC104-700	POP104	Pyrolysis	700 °C
9	NPC101-900	POP101	Pyrolysis	900 °C
10	NPC102-900	POP102	Pyrolysis	900 °C

**Table 2:** Surface area, pore size distribution, and pore volumes of the synthesized POPs and NPCs.

Material	BET Area (m^2^ g^−1^)	Langmuir Area (m^2^ g^−1^)	Pore Volume ^1^ (cm^3^ g^−1^)	Micropore Volume ^2^ (cm^3^ g^−1^)	DFT Pore Radius (nm)
POP101	205	324	0.59	0.000	0.25
POP102	263	441	0.62	0.000	0.62
POP103	333	532	0.50	0.000	0.59
POP104	436	695	0.55	0.010	0.63
NPC101-700	570	792	0.60	0.201	0.39
NPC102-700	369	552	0.42	0.094	0.39
NPC103-700	387	542	0.34	0.080	0.54
NPC104-700	348	495	0.30	0.065	0.54
NPC101-900	537	807	0.78	0.141	0.39
NPC102-900	659	1067	0.83	0.120	0.50

^1^ DFT accumulated pore volume. ^2^ Micropore volume calculated using t-plot method, thickness method: DeBoer.

**Table 3:** Data of all CO_2_ adsorption capacities at 273 K and 298 K.

Samples	CO_2_ Uptake (cm^3^ g^−1^)	CO_2_ Uptake (cm^3^ g^−1^)
	273 K	298 K
POP101	30.8	20.7
POP102	29.3	19.2
POP103	28.9	19.3
POP104	30.8	19.6
NPC101-900	59.4	44.1
NPC102-900	57.5	46.5

**Table 4:** Thermodynamic parameters were calculated for NPC101 and NPC102.

Material	ΔS° (kJ/mol K)	ΔH° (kJ/mol)	ΔG° (kJ/mol)	
			273 K	298 K
NPC101-900	−0.074	−23.620	−3.35	−1.50
NPC102-900	−0.044	−14.827	−2.73	−1.62

**Table 5:** Comparison table of NPCs’ performance with similar related material toward CO_2_ capture.

Material	SA_BET_ (m^2^ g^−1^)	CO_2_ Uptake (mmol g^−1^)	Temp. (K)	CO_2_/N_2_ Selectivity	Ref.
POP-derived NPCs	570–659	1.9–2.1	298	50–53	This work
CMK-3 carbon	624	1.7	293	35–38	[42]
Mesoporous carbon-MgO		0.9	298	—	[43]
Mesoporous N-doped carbon tea waste (TW-900)	354	1.7	298	79	[44]
Activated porous biocarbons (NEPB-3UK)	982	2.2	298	—	[45]
BPL Carbon	1210	2.1	298	—	[46]
Cellulose-based carbons -AC-N_2_	500	2.6		—	[47]
benzoate-derived porous carbon	777	3.6	298	—	[48]
(N, S, O, P)-doped porous carbon microspheres (PCMS—750)	342	1.8	298	10.6	[49]
PCMSs—800	481	2.8		12.7	[49]
Hierarchical porous carbon		4.5	273	—	[50]
Carbon monoliths	595–621	2.3–3.0	298	—	[51]

## Data Availability

Not applicable.
